# High CYP27A1 expression is a biomarker of favorable prognosis in premenopausal patients with estrogen receptor positive primary breast cancer

**DOI:** 10.1038/s41523-021-00333-6

**Published:** 2021-09-23

**Authors:** Maria Inasu, Pär-Ola Bendahl, Mårten Fernö, Per Malmström, Signe Borgquist, Siker Kimbung

**Affiliations:** 1 Division of Oncology, Department of Clinical Sciences Lund, Lund University, Lund, Sweden; 2 Department of Hematology, Oncology and Radiation Physics, Skåne University Hospital, Lund, Sweden; 3 Department of Oncology, Aarhus University and Aarhus University Hospital, Aarhus, Denmark

**Keywords:** Prognostic markers, Breast cancer, Tumour biomarkers, Cancer epidemiology

## Abstract

27-hydroxycholesterol (27HC), synthesized from cholesterol by the enzyme CYP27A1, differentially impacts estrogen receptor positive (ER+) breast cancer (BC) cell growth depending on estrogen levels. This study examined the association between CYP27A1 expression and prognosis in a cohort of 193 premenopausal patients with lymph node-negative primary BC with limited exposure to adjuvant systemic cancer treatments. In multivariable analyses among patients with ER+ tumors, high CYP27A1 protein and mRNA expressions were associated with four- and eight-fold reductions in the incidence of distant recurrence-free survival events: HR_adj_ = 0.26, 95% CI = 0.07–0.93 and HR_adj_ = 0.13, 95% CI = 0.03–0.60, respectively. In vitro studies revealed that 27HC treatment potently inhibited ER+ BC cell proliferation under lipid-depleted conditions regardless of estradiol levels, transcriptionally mediated through the downregulation of ER signaling with a concomitant upregulation of cholesterol export. Importantly, if validated, these results may have implications for adjuvant treatment decisions in premenopausal patients, especially when de-escalation of therapy is being considered.

## Introduction

Breast cancer (BC) is characterized by marked heterogeneity at the molecular and morphological levels, dictating varied prognosis and requiring diverse therapeutic interventions. BC is classified into distinct clinical subtypes based on the expression of hormone receptors and human epidermal growth factor receptor 2 (HER2)^1,2^. Luminal, estrogen receptor positive (ER+) disease is the most common subtype^3^ and is driven by direct interactions between the ligand estrogen and its cognate receptor, ER, that leads to the transcriptional activation of genes central to cell growth and survival^4^. The crux of systemic treatment of patients with ER+ BC relies on drugs that either prevent the synthesis of the estrogen ligand by inhibiting the aromatase enzyme, competitively inhibiting ligand binding, or degrading the cellular receptor for the ligand^5^. Despite adjuvant endocrine treatments, 20–30% of patients still experience disease recurrence^6,7^. Moreover, because of the lack of accurate treatment predictive and prognostic biomarkers, over-treatment of patients remains a clinical challenge, as all treatments are associated with undesirable side effects^8^. Consequently, there is a constant search for novel biomarkers to further refine prognostication and tailor treatments on an individual basis if possible.

The mounting pre-clinical evidence that 27-hydroxycholesterol (27HC), an oxysterol synthesized from cholesterol by the cytochrome p450, family 27, subfamily A polypeptide 1 (CYP27A1), is a selective estrogen receptor modulator (SERM), has partly explained the biochemical link between cholesterol and BC^9–12^. 27HC is a SERM that behaves as a partial agonist in ER+ BC models. However, being also an oxysterol, 27HC can bind to and activate liver x receptor (LXRs). The extent/function of downstream signaling via each receptor is, however, dependent on the specific cellular context^13^. In the absence of estrogen, 27HC can stimulate ER+ BC growth in vitro and in mice through direct interactions with ER, driving the transcription of genes necessary for cell growth and survival^9–12^. In the presence of estrogen, 27HC, however, competes with estrogen for the ligand-binding site on the ER, antagonizing the proliferative effects of estrogen on ER+ BC cells in vitro^9,12^. Importantly, this estrogen dependency of 27HC activation of the ER qualifies it as the only characterized naturally occurring SERM, with a potential to differentially impact the development and outcome of clinical BC depending on the patient’s menopausal status. It remains to be shown whether the effects of 27HC on ER+ BC proliferation are also dependent on other factors such as the levels of other lipids in the surroundings. To develop the optimal approach for targeting 27HC in BC, a better understanding of all factors that impact 27HC function is necessary.

It has been reported that, on average, 27HC levels are higher in serum from BC patients compared to women without BC and that the average bioavailability of 27HC in tumors is higher compared to normal breast tissue^11^. Furthermore, 27HC levels vary significantly among breast tumors, and *CYP27A1* is also differentially expressed among tumors from different patients^10,11,14^ and this variability may have implications for prognosis and treatment. Considering that CYP27A1 is responsible for regulating 27HC levels, it is reasonable to assume that the differential CYP27A1 expression may serve as a surrogate biomarker for 27HC levels in the tumors. The advantage of such a proxy biomarker is that it can be assessed conveniently by assays like immunohistochemistry (IHC) and in situ RNA hybridization techniques compared to mass spectrophotometry, which is the current gold standard for measuring 27HC requiring complex equipment and large amounts of breast tumor tissue^14,15^.

The prognostic impact of CYP27A1 expression in BC has been previously investigated. Tumoral *CYP27A1* mRNA expression was initially found to be correlated with increased recurrence free survival time in BC patients^13,14^. However, this favorable outcome was shown to be restricted to the subgroup of patients under 50 years of age presenting with ER+ tumors^14^. Elevated expression of CYP27A1 protein was recently observed to be a marker of late lethal disease in a large cohort of postmenopausal BC patients^16^. The prognostic impact of CYP27A1 in premenopausal patients is currently controversial. In contrast with the observation that high *CYP27A1* mRNA expression was associated with a favorable prognosis compared to low *CYP27A1* mRNA expression among the BC patients ≤50 years^14^, high CYP27A1 protein expression was found to be associated with inferior prognosis compared with low CYP27A1 protein expression among women <55 years old in a later study^16^, warranting more data, preferably from better characterized and larger cohorts.

Based on previously validated in vitro mechanistic data^9–11^ and the preliminary clinical evidence^14^, we hypothesized that high expression of CYP27A1 is associated with a better prognosis in premenopausal patients with ER+ breast cancer. The primary aim of this study was to evaluate the independent prognostic impact of CYP27A1 protein and mRNA expression in a well characterized cohort of premenopausal BC patients. Next, we aimed to investigate whether in the absence of other serum lipids, 27HC alone was sufficient to impact ER+ cell growth in an estrogen-rich milieu, and whether the effects, if any, were mediated via modulating ER signaling and cellular cholesterol homeostasis.

## Results

### Patients and tumor characteristics in relation to CYP27A1

Generally, patient and tumor characteristics had similar distributions between the overall cohort (*n* = 237) and the study-specific subset of patients (*n* = 193) who were evaluable for both CYP27A1 protein and mRNA expression (Supplementary Table 1). For the study-specific cohort, the median age was 47 years (range 29–56). A total of 74.6% of the tumors were T1 ( ≤20 mm), 66.4% were Nottingham histological grade (NHG) I or II, 66.3% and 71.5% were ER and progesterone receptor (PgR) positive, respectively, 87.2% were HER2 negative, and 66.1% had low Ki67 expression. Regarding adjuvant systemic treatments, only 9.3% and 4.1% received chemotherapy and endocrine therapy, respectively (Supplementary Table 1). High CYP27A1 expression of both protein and mRNA were associated with adverse tumor pathological characteristics such as NHG III (*P=* 0.002 and *P* < 0.001, respectively), and high Ki67 expression (*P* < 0.001 for both) (Table 1). CYP27A1 expression was not differentially associated with age, tumor size, ER, PgR, or HER2 (Table 1).

**Table 1:** Distribution of tumor pathological features according to CYP27A1 expression in 193 breast cancer patients.

	CYP27A1 (protein)			*CYP27A1* (mRNA)		
	Low (%)	High (%)	*P*	Low (%)	High (%)	*P*
Age^a^ (continous)	46.8 (29.7– 56.3)	46.8 (30.2–53.2)	0.52	46.9 (29.7–56.3)	46.5 (30.2–52.1)	0.43
Tumor size, mm						
≤20	101 (73.7)	43 (76.8)	0.66	110 (74.3)	34 (75.6)	0.87
>20	36 (26.3)	13 (23.2)		38 (25.7)	11 (24.4)	
Nottingham histological grade						
I and II	104 (74.2)	27 (50.9)	0.002	110 (74.8)	19 (43.2)	<0.001
III	36 (25.7)	26 (49.0)		37 (25.2)	25 (56.8)	
ER status						
Positive	90 (65.7)	38 (67.9)	0.77	100 (67.6)	28 (62.2)	0.51
Negative	47 (34.3)	18 (32.1)		48 (32.4)	17 (37.8)	
PgR status						
Positive	100 (73.0)	38 (67.9)	0.47	106 (71.6)	32 (71.1)	0.95
Negative	37 (27.0)	18 (32.1)		42 (28.4)	13 (28.9)	
HER2 status						
Positive	18 (14.3)	5 (9.4)	0.38	18 (13.3)	5 (11.4)	0.76
Negative	108 (85.7)	48 (90.6)		117 (86.7)	39 (88.6)	
Ki67, %						
≤20	91 (75.2)	24 (45.3)	<0.001	96 (73.3)	19 (44.2)	<0.001
>20	30 (24.8)	29 (54.7)		35 (26.7)	24 (55.8)	

^a^Median (Range)

### Correlations between CYP27A1 protein and mRNA expression

Inter-reader agreement for the dichotomized CYP27A1 expression score was high for both IHC (kappa (κ) = 0.93) and RNAscope (κ = 0.85), respectively. A moderate intratumoral concordance of 77.7% (r_s_ = 0.43) was observed between CYP27A1 protein and mRNA expression. One hundred and twenty-one tumors (62.7%) displayed concordant low expression and 29 tumors (15%) showed concordant high expression. Sixteen tumors (8.3%) had low protein/high mRNA expression and 27 tumors (14%) had high protein/low mRNA expression.

### Prognostic impact of CYP27A1 protein expression

**Fig. 1:**
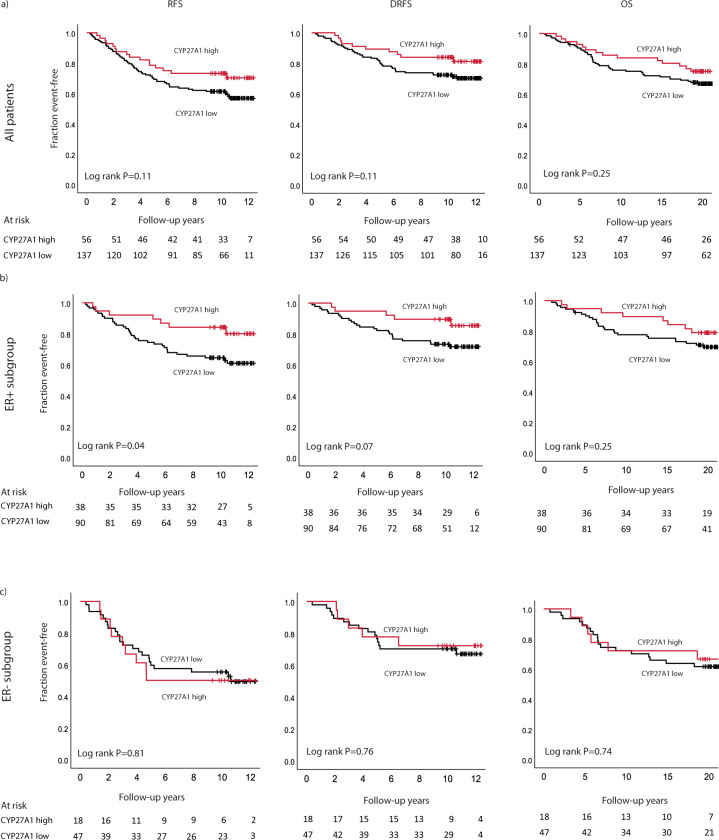
Kaplan–Meier graphs showing associations between CYP27A1 protein expression and survival. **a** All patients, **b** patients with ER+ tumors, and **c** patients with ER– tumors.

All patients were followed for a median of 10 years (0.3–12.4) for any event of BC recurrence and 20 years (0.7–22.4) for overall survival (OS). At the end of follow-up, 73 recurrence-free survival (RFS), 50 distant recurrence-free survival (DRFS), and 59 OS (of which 49 are BC specific deaths) events had been recorded. High CYP27A1 expression was associated with a statistically non-significant trend for a longer event-free survival compared to low CYP27A1 expression, for all patients as well as for patients with ER+ tumors (Fig. 1a, b). Similar trends were observed in univariable Cox regression analyses (DRFS; HR = 0.57, 95% CI = 0.28–1.14 and HR = 0.43, 95% CI = 0.16–1.12, for all tumors and ER+ tumors, respectively). No difference in overall survival according to CYP27A1 expression was observed among ER negative patients (Fig. 1c).

Notably, after adjusting for patient age, tumor size, ER, HER2, NHG, Ki67 and adjuvant treatments in multivariable analysis, high CYP27A1 protein expression emerged as a strong and independent prognostic factor for a better DRFS among patients with ER+ tumors, associated with a 74% decrease in the incidence of DRFS events (HR_adj = _0.26, 95% CI = 0.07–0.93). Among all patients, the incidence of DRFS events decreased by 58% (HR_adj = _0.42, 95% CI = 0.18–1.01), although statistically insignificant. Similar trends toward a better prognosis were observed for the endpoints RFS and OS (Table 2).

**Table 2:** Multivariable-adjusted hazard ratios (95% CI) for all evaluated endpoints by tumor CYP27A1 expression.

			Hazard ratio (95% CI)			
	No of patients	No of events	CYP27A1 protein^a^*	CYP27A1 protein^b^**	*CYP27A1* mRNA^a^*	*CYP27A1* mRNA^b^**
Recurrence-free survival						
All	164^#^	61	0.42 (0.21–0.84)	0.42 (0.21–0.85)	0.38 (0.18–0.78)	0.37 (0.18–0.78)
			*P* = 0.01	*P* = 0.02	*P* = 0.009	*P* = 0.009
ER+	108	34	0.20 (0.06–0.8)	0.18 (0.06–0.56)	0.22 (0.07–0.66)	0.19 (0.06–0.57)
			*P* = 0.003	*P* = 0.003	*P* = 0.007	*P* = 0.003
ER−	56	27	1.04 (0.36–3.00)	1.10 (0.37–3.26)	0.66 (0.22–1.94)	0.79 (0.24–2.64)
			*P* = 0.94	*P* = 0.86	*P* = 0.45	*P* = 0.70
Distant recurrence-free survival						
All	164	42	0.39 (0.17–0.93)	0.42 (0.18–1.01)	0.25 (0.09–0.67)	0.26 (0.10–0.72)
			*P* = 0.03	*P* = 0.05	*P* = 0.006	*P* = 0.01
ER+	108	24	0.25 (0.07–0.89)	0.26 (0.07–0.93)	0.15 (0.03–0.66)	0.13 (0.03–0.60)
			*P* = 0.03	*P* = 0.04	*P* = 0.01	*P* = 0.009
ER−	56	18	0.65 (0.15–2.74)	0.64 (0.15–2.66)	0.45 (0.11–1.87)	0.44 (0.09–2.18)
			*P* = 0.56	*P* = 0.54	*P* = 0.27	*P* = 0.31
Overall survival						
All	164	49	0.52 (0.25–1.08)	0.53 (0.25–1.14)	0.21 (0.08–0.56)	0.21 (0.08–0.56)
			*P* = 0.08	*P* = 0.10	*P* *=* 0.002	*P* = 0.002
ER+	108	28	0.40 (0.15–1.10)	0.40 (0.14–1.12)	0.13 (0.03–0.59)	0.12 (0.02–0.54)
			*P* = 0.07	*P* = 0.08	*P* = 0.008	*P* = 0.006
ER−	56	21	0.70 (0.20–2.42)	0.65 (0.17–2.46)	0.33 (0.09–1.26)	0.37 (0.09–1.65)
			*P* = 0.57	*P* = 0.52	*P* = 0.11	*P* = 0.19

Hazard ratios compare CYP27A1 high to CYP27A1 low.

*Model a: Model adjusted for age at diagnosis, tumor size, Ki67, ER, HER2, Nottingham histological grade.

**Model b: Model a + adjusted for local (radiotherapy) and systemic (endocrine and chemotherapy) treatment.

^#^29 cases had missing value for at least one of the adjusted variables.

### Prognostic impact of *CYP27A1* mRNA expression

**Fig. 2:**
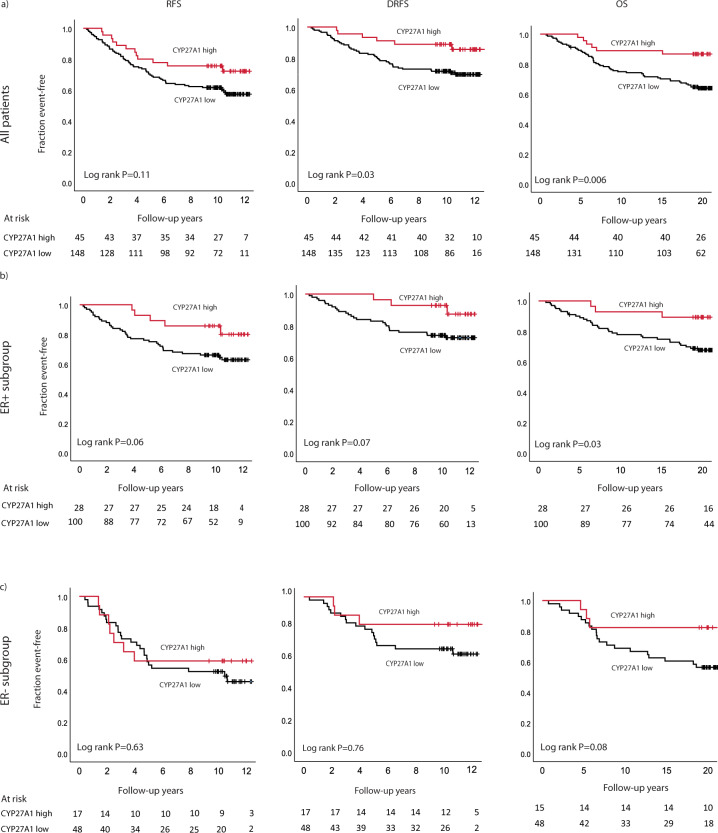
Kaplan–Meier graphs showing associations between CYP27A1 mRNA expression and survival. **a** All patients, **b** subset of patients with ER+ tumors, and **c** ER– tumors.

Next, the prognostic impact of *CYP27A1* mRNA expression was assessed among all patients and in the pre-specified subgroup of patients with ER+ tumors in univariable analysis. Among all patients, high *CYP27A1* expression was significantly associated with a better prognosis compared to low *CYP27A1* expression for the endpoints DRFS and OS (Fig. 2a). Similarly, among patients with ER+ tumors, a borderline trend towards longer event-free survival was noted for tumors with high *CYP27A1* mRNA expression (Fig. 2b). No difference in survival was seen between *CYP27A*1 low vs high tumors among patients with ER negative tumors (Fig. 2c).

Remarkably, after adjusting for all available tumor-pathological factors in multivariable models (Table 2), high *CYP27A*1 expression was independently and strongly prognostic for longer DRFS (HR_adj = _0.26, 95% CI = 0.10–0.72) for all patients and for the subset of patients with ER+ tumors, DRFS (HR_adj = _0.13, 95% CI = 0.03–0.60). Similar strong effects were seen also for the endpoints RFS and OS (Table 2).

### Impact of differential CYP27A1 protein and mRNA expression on prognosis

To explore if the intra-tumoral differential expression between CYP27A1 protein and mRNA impacted outcome, we performed a multivariable analysis considering the double negative CYP27A1 expression (mRNA−/protein−) as the reference group. The combination of high CYP27A1 mRNA and protein expression (mRNA+/protein+) was significantly associated with longer survival compared with the double negative CYP27A1 expression (mRNA−/protein−) for all end points (*P* < 0.05 for all comparisons, Supplementary Table 2). Comparable results were noted for the mRNA expression alone (mRNA+/protein−), although with weaker statistical significance. Protein expression alone (mRNA−/protein+) trended similarly with a non-significant association to longer event-free survival compared to the double negative group (Supplementary Table 2).

### Prognostic importance of CYP27A1 in relation to NHG and Ki67

**Fig. 3:**
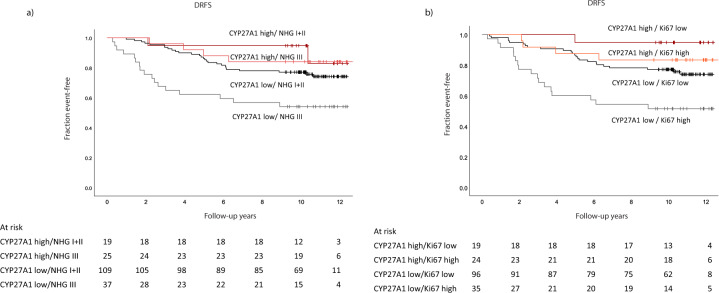
Kaplan–Meier graphs showing the joint prognostic impact of CYP27A1 expression with NHG and Ki67. **a** CYP27A1/NHG, **b** CYP27A1/Ki67.

The strong positive associations between CYP27A1 expression with aggressive tumor characteristics like NHG III and high Ki67 and the finding that high CYP27A1 is associated with favorable prognosis (Tables 1 and 2) are perplexing and prompted us to explore the joint impact of these markers on prognosis. As depicted in the Kaplan–Meier graphs in Fig. 3, high *CYP27A1* mRNA expression identified a subset of tumors within both the Ki67 high and NHG III group that displayed a more favorable prognosis relative to the rest of the tumors in these otherwise poor prognostic subgroups. Importantly, high CYP27A1 expression was prognostic within both the high and low Ki67 categories and across NHG groups. In multivariable Cox regression analyses, weak evidence for better prognosis was seen for tumors with elevated *CYP27A1* despite the high expression of proliferation markers (Supplementary Table 3).

### Effect of 27HC treatment on ER+ BC cells cultured in lipid-depleted medium supplemented with estradiol

**Fig. 4:**
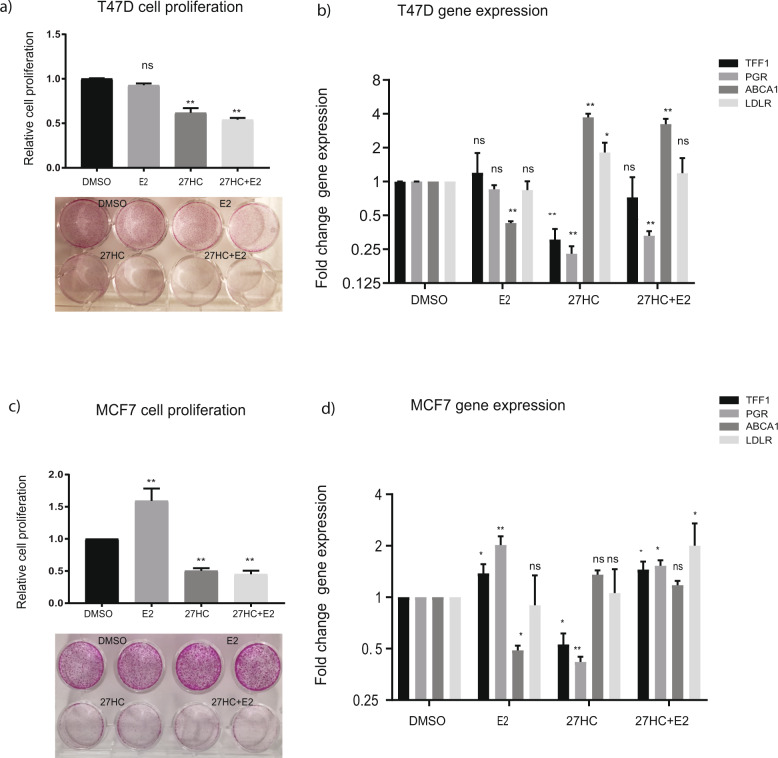
Effects of 27HC treatment on ER+ breast cancer cells under lipid-depleted conditions. T47D and MCF7 cells were treated with 1μM 27HC, 1nM of E2, or a combination of both for 72 h and proliferation measured using SRB assay. **a**, **c** The quantification of cell proliferation for T47D and MCF7 respectively under respective treatment conditions. These panels also feature a representative image of post treatment SRB stained cells. After treating the cells under respective conditions, changes in expression of ER target genes TFF1, PgR, and LXR target genes ABCA1, LDLR was measured by qRT-PCR. **b**, **d** The fold change in gene expression in T47D and MCF7 cells, respectively. Quantifications are presented as fold change compared to vehicle DMSO treatment. All data are shown as the mean +/– SD of at least two representative experiments. *p < 0.05, **p < 0.01.

Following the observation that high CYP27A1 expression predicts better prognosis in premenopausal patients with ER+ BC, we next explored in vitro whether the growth inhibitory effects of 27HC in the presence of estrogen was dependent on the availability of other serum lipids. For this purpose, we selected two luminal (ER + /PgR + /HER2−) and estrogen dependent cell lines T47D and MCF7 that are commonly used to study BC biology and response to endocrine therapies in vitro. Treatment of T47D and MCF7 cells with 27HC alone under lipid-depleted conditions potently inhibited cell proliferation by 38% in T47D and 50% in MCF7 relative to the vehicle (DMSO) control [T47D; *P* = 0.002, MCF7; *P* < 0.001 (Fig. 4a, c)]. Conversely, the addition of estradiol alone to the lipid-depleted medium resulted in a 59% increase in MCF7 cell proliferation (*P* = 0.001) relative to DMSO control. The addition of estradiol (only) did not significantly impact the basal proliferative rate of T47D cells (*P* = 0.12). Interestingly, the addition of estradiol did not rescue the cells from the potent anti-proliferative effects induced by 27HC in both cell lines (T47D; *P* = 0.001, MCF7; *P* < 0.001) (Fig. 4a, c).

Next, we characterized the molecular changes induced by 27HC treatment under the same conditions by evaluating changes in two key cellular processes that can potentially be impacted by 27HC: estrogen receptor signaling and cholesterol homeostasis. To address this, the expression of two candidate ER downstream target genes (*TFF1* and *PgR*) and two genes involved in intracellular cholesterol export (*ABCA1*) and import (*LDLR*), respectively, were measured. Treatment with 27HC alone downregulated the expression of *TFF1* and *PgR* in both cell lines. Conversely, a significant upregulation of *ABCA1* was noted in T47D cells when 27HC treatment was provided. However, *ABCA1* expression was not impacted by 27HC treatment in MCF7 cells. Furthermore, estradiol upregulated the expression of both ER target genes only in MCF7, aligning with the observed increase in cell proliferation under this condition. *ABCA1* expression was also significantly downregulated in both cell lines in the presence of estradiol only (Fig. 4b, d). Overall, under lipid-depleted conditions, the effects of 27HC treatment on cell proliferation were similar in both T47D and MCF7 cells regardless of the presence of estradiol. While T47D cells displayed similar gene expression trends in the presence of 27HC regardless of estradiol presence, the effects of 27HC on gene expression in MCF7 seemed to vary with estradiol availability.

## Discussion

In this study, we have evaluated the independent impact of CYP27A1 protein and transcript expression on the prognosis of primary invasive BC among premenopausal patients with lymph node-negative disease. This study is based on a cohort of patients from the South Swedish Health Care Region who were diagnosed with BC between 1991 and 1994. High intratumoral CYP27A1 expression was found to be associated with a better prognosis after adjusting for potential confounding factors. Specifically, the incidence of disease recurrence and/or death among patients presenting with ER+ BC was reduced by more than a factor of two for patients with CYP27A1 high tumors compared to patients with CYP27A1 low tumors.

The strong epidemiological evidence showing the protective effect of cholesterol-lowering medication against BC recurrence^17–20^ and the recent pre-clinical experimental evidence linking cholesterol to BC growth^10,11,21^ have increased the interest in targeting cholesterol metabolism to delay or prevent BC recurrence. 27HC, being one of the biochemical links that directly connects cholesterol to BC cell proliferation, is a top candidate for therapeutic development. However, because of the limitations of the current methods for quantifying 27HC which requires large amounts of tumor material, the importance of 27HC in clinical BC is not well established. Alternative biomarkers like CYP27A1 have been used to investigate links between cholesterol metabolism and clinical BC prognosis. We and others have previously shown that CYP27A1 is differentially expressed between tumors and may be prognostic in some cancer types^10,14,22–24^. Specifically in BC, the prognostic impact of *CYP27A1* mRNA expression was previously studied in patients (not stratified for age or menopausal status) with one study showing a null association with RFS^10^ while another study showed an association between high *CYP27A1* mRNA expression with improved RFS^13^. However, when patients were stratified by hormone receptor expression and age at diagnosis in a subsequent large study^14^, high expression of *CYP27A1* mRNA was found to be specifically associated with improved RFS and OS among patients younger than 50 years presenting with ER+ disease only, lending clinical support to the preclinical experimental evidence that 27HC antagonizes estrogen-induced BC growth. In contrast, in a recently conducted IHC-based study^16^, high CYP27A1 protein expression was conversely associated with a higher incidence of disease recurrence among patients younger than 55 years with ER+ tumors, raising questions about the true significance of CYP27A1 in premenopausal BC. Thus, by evaluating the expression of both CYP27A1 mRNA and protein expression in the same tumor series from premenopausal patients, this study has demonstrated that intratumoral CYP27A1 transcript and protein expression are moderately correlated and that high protein and mRNA expression of CYP27A1 are both prognostic of a favorable outcome in premenopausal patients with ER+ BC, albeit to a varying extent. Importantly, our results suggest that *CYP27A1* transcript expression may perform better than protein expression as a biomarker for prognostication purposes, for all endpoints evaluated in this study (similarly high CYP27A1 expression was also associated with a better breast cancer specific survival among all patients and subgroups; data not shown). The strength of the *CYP27A1* mRNA biomarker was further exemplified by its ability to differentiate the prognosis not only among subgroups like patients with NHG III and high Ki67, which are typically considered to be of “poor” prognosis, but even among subgroups like patients with NHG I & II and low Ki67, which are typically considered to be of “good” prognosis. These findings highlight the potential of *CYP27A1* as a biomarker to aid in decisions about treatment avoidance or de-escalation for premenopausal ER+ BC patients, warranting further validation studies.

Based on the concordant associations between CYP27A1 protein and mRNA expression and premenopausal ER+ BC prognosis observed in this study, the contradictory results reported in the former IHC-based study^16^ may be likely attributed to the more generous use of systemic adjuvant therapies like chemotherapies and/or ovarian suppression treatments. These treatments are known to decrease estrogen levels^25–27^, resulting in a milieu where 27HC might exert pro-proliferative effects instead of anti-proliferative effects and, therefore, leading to a higher disease recurrence rate among the patients with high CYP27A1 tumors compared with low CYP27A1 tumors. Moreover, tamoxifen is the standard adjuvant endocrine treatment offered to younger patients. Tamoxifen is a SERM and has been shown to impact cholesterol levels in circulation^17^. Treatment with tamoxifen theoretically would create an even complex situation where three different ligands (tamoxifen, 27HC and estrogen) would be competing to bind to the estrogen receptor. Previously Nelson et al.^10^ demonstrated that 27HC can even sustain the growth of tamoxifen-resistant MCF7 xenografts in ovariectomized, immunocompromised mice. Interestingly, the use of adjuvant chemotherapy and endocrine therapies was also limited in the primary study that showed a favorable prognostic impact of *CYP27A1* mRNA among women under 50 years old^14^. The prognostic impact of CYP27A1 in the context of conventional adjuvant treatments and the effects of combining 27HC with modern BC treatments including targeted therapies needs to be investigated in preclinical and better sized clinical studies.

Notably, in this study of premenopausal patients with lymph node negative BC, the use of systemic adjuvant endocrine and chemotherapies was minimal (9.3% and 4.1% of patients that had received chemotherapy and endocrine therapy, respectively). This provided a unique opportunity to evaluate, to a large extent, the “natural” prognostic impact of CYP27A1 (27HC). Multivariable analyses excluding all the 26 patients who received any kind of adjuvant systemic treatment yielded very similar results for all study endpoints and subgroups, especially for the *CYP27A1* mRNA biomarker (data not shown). Our results highlight the good potential of high CYP27A1 expression to be another biomarker for favorable prognosis, specifically useful in identifying a small subset of premenopausal BC patients with ER+ disease who may be exempted from adjuvant treatment. It is, however, not clear what class of adjuvant systemic treatments may be candidates for therapy de-escalation in these patients considering that under the current clinical guidelines, most of the patients in this cohort would be recommended for at least 5 years of an adjuvant endocrine therapy. Of note, until a direct correlation between CYP27A1 expression and intratumoral 27HC levels has been established, CYP27A1 expression may not be interpreted to reflect the concentrations or biology of 27HC. It has been reported that CYP7B1 (the enzyme responsible for catabolizing 27HC) expression and not CYP27A1 correlates better with 27HC levels in thyroid cancer^28^ and CYP7B1 has been shown to influence immune function in tumor progression and associated to BC prognosis^10,13^.

In this study, we have specifically investigated in vitro whether the previously reported antagonistic effects of 27HC on estrogen induced ER+ cell proliferation is sustained in the absence of other serum lipids. In order to design an optimal approach for targeting 27HC in BC, it is important to fully understand how other surrounding factors impacts it’s activity. Our results, though preliminary only, show that 27HC potently inhibits estrogen induced ER+ BC cell growth even in the absence of other serum lipids, demonstrating the specificity of the effect to 27HC. The observed antiproliferative effects of 27HC in the absence of lipids may suggest that for a better understanding of the clinical impact, it may be necessary to consider other patient and tumor characteristics associated with high lipid levels such as hyperlipidemia, obesity, intra-tumoral lipid content in predicting prognosis.

The reported range of 27HC concentration in serum from healthy individuals is 0.15–0.73 µM^12^ and 0.15–0.90 μM in BC patients^13,14^. 27HC is reported to reach millimolar levels in developing foam cells and atherosclerotic plaques^12^. However, the average concentration of 27HC in clinical breast tumors has not been satisfactorily estimated. For in vitro investigations, we have used 1 µM of 27HC, a concentration that was shown to be sufficient to drive the proliferation of ER+ cells grown in cell culture medium supplemented with the estrogen-free (but not lipid-depleted) charcoal-stripped fetal bovine serum (FBS)^9,10^. Hence, the potent anti-proliferative effect observed in this study is attributable to 27HC, but whether this concentration of 27HC and the corresponding effects are clinically achievable intratumorally remains to be determined. Further characterization of the response at the molecular level established that the anti-proliferative effects were associated with a commensurate downregulation of ER signaling and upregulation of cholesterol efflux, especially in T47D cells. It has been firmly established that being a SERM, 27HC is capable of downregulating ER signaling in the presence of estradiol^9,12^, but the effects under lipid-depleted conditions were previously unknown. Of the nuclear receptors, the liver-X receptor (LXR) is thought to be the principal target of oxysterols, especially when considering the regulation of intracellular cholesterol metabolism. Upregulation of the cholesterol transporters *ABCA1* and *ABCG1* is mediated by the activation of LXR upon 27HC binding^29^. Oxysterol activation of the LXR can also antagonize BC cell proliferation^30–32^. We found that 27HC treatment induced the expression of the cholesterol exporter *ABCA1* even in the presence of sufficient estradiol in T47D and not in MCF7 cells. This disparate finding continues to highlight the remarkable heterogeneity of BC, especially at the molecular level. Despite the diverse response to treatment displayed at the gene expression level between the cell lines, the potent anti-proliferative effect of 27HC was consistent between the cell lines, indicating that the expected phenotype after 27HC treatment under these conditions is reduced cell growth. Our in vitro results are only preliminary findings and should be interpreted with caution. Though unlikely that a tumor/patient will ever experience the lipid depleted conditions as in our cell culture experiments, the results nonetheless reveal some novel and important information about the activity of 27HC in ER+ BC and open the interesting question of whether factors such as lipid/cholesterol levels in circulation and the tumor microenvironment impact the prognostic significance of 27HC (CYP27A1) on clinical ER+ BC prognosis in addition to menopausal status.

Today, biomarkers that predict favorable outcomes requiring minimal therapeutic interventions are highly attractive. We have identified a biomarker that can be measured using relatively convenient assays and which has a good potential to identify a subset of patients with ER+ lymph node-negative BC who have a favorable outcome following limited or no systemic adjuvant treatment. In the current clinical practice, these patients with high CYP27A1 tumors may be candidates for de-escalating systemic adjuvant therapies or even omission of adjuvant treatment. Given that oncological therapy is always associated with some degree of toxicity, it remains a priority to continue searching for and validating novel prognostic and treatment predictive biomarkers that will support clinical decision-making regarding therapy choices for individual patients. Future studies should therefore investigate the prognostic impact of CYP27A1 expression in larger cohorts of premenopausal BC patients who have received adjuvant endocrine therapy alone.

## Methods

### Patients and tumor microarray construction

The study population consists of a series of 237 premenopausal patients with lymph-node-negative BC included from 1991 to 1994 in a prospective clinical trial (SB91B) conducted to validate the prognostic value of an index based on tumor proliferation (S-phase fraction), PgR status, and tumor size^33,34^. All patients underwent radical operation for early breast cancer with either a standardized sector resection or a modified radical mastectomy. Dissection of levels 1 and 2 of the axilla was performed in all patients. A median number of nine nodes were identified in the resected axillary specimen. Patients treated with breast-conserving surgery were randomized to either postoperative radiotherapy (49.4%) 50 Gy in 25 fractions to the remaining breast parenchyma or to no further local treatment (50.6%) in a Swedish multicenter trial evaluating postoperative radiotherapy. Archival formalin-fixed paraffin-embedded (FFPE) blocks were retrieved for 223 cases from the pathology department. For tumor microarray (TMA) construction, representative tumor cell areas on the FFPE blocks were selected and two 1.0 mm cores were obtained and transferred onto the recipient TMA blocks. Sections of 3–4 µm were cut and mounted onto a glass slide for CYP27A1 evaluation. All patients provided their informed consent to be included in the SB91B clinical trial. The study was approved by the ethics committee of Lund University Hospital (LU 240-01).

### NHG, ER, PgR, HER2, and Ki67 evaluation

Tumor pathological assessment for this cohort has been described earlier^33,34^. Briefly, tumor histological grading was assessed according to Elston and Ellis^35^. ER and PgR were analysed in the cytosol fraction by enzyme immunoassay (Abbott Laboratories, Diagnostic Division, Chicago, IL, USA). HER2 gene amplification was assessed by the FISH pharmDxTM Kit (DAKO K5331, Copenhagen, Denmark) and Ki67 expression was quantified by IHC using the antibody MIB-1 (DAKO, K5001, Copenhagen, Denmark).

### CYP27A1 protein expression

CYP27A1 protein expression was measured by IHC following a previously validated protocol^10,14,16,22^. Briefly, tumor sections were de-paraffinized, treated with antigen retrieval buffer for 20 min, and then reacted with an anti-CYP27A1 rabbit monoclonal antibody (Abcam, [EPR7529], ab126785) at a dilution of 1:300 for 2 h. CYP27A1 expression was evaluated in tumor cells only and the staining was quantified using a semi-quantitative scale: a tumor was considered to express CYP27A1 only if ≥20% of the tumor cells in all evaluable cores per tumor (patient) showed a granular cytoplasmic antigen-antibody reactivity. Furthermore, the staining was categorized into four intensity levels [0 (absent), 1 (weak), 2 (moderate), or 3 (strong)]. For statistical analyses, a dichotomized score was used; 0–1 (low) or 2–3 (high), consistent with previous studies^10,14,16,22^. Two investigators (MI and SK) blinded to other pathological data assessed the IHC staining. Inter-reader agreement for the dichotomized score was high, kappa (κ) = 0.93, with only six discordant tumors. For discordant cases, a final score was assigned following a consensus from both investigators. Finally, due to the lack of archival FFPE blocks, missing cores, non-intact or poor-quality cores, or the absence of tumor cells in the core, CYP27A1 protein expression was evaluable for 196 tumors. The REMARK guidelines were respected in the biomarker assessment and analyses presented in this study^36^.

### *CYP27A1* mRNA expression

*CYP27A1* transcript levels were quantified by RNAscope according to the process manufacturer´s guidelines (ACDbio)^37^. Tumor tissue sections on slides were de-paraffinized with xylene and ethanol, then treated for 15 min with target retrieval buffer (RNAscope^®^ Target retrieval reagent, Cat#322000). Sections were covered with custom-made *CYP27A1* target probes (RNAscope^®^ Probe- Hs-CYP27A1, Cat#548601) for two hours at 40 °C, washed, and subsequently treated for 15 to 30 min with signal amplification reagents (RNAscope^®^ 2.5 HD AMP1-6, Cat#322310). Diaminobenzidine (RNAscope^®^ 2.5 HD DAB-A, B, Cat# 322310) was used as the chromogen and hematoxylin to counterstain the cell nuclei. To analyze the target gene expression a semi-quantitative scoring was followed; 0 = No staining or <1 dot to every 10 cells; 1 = 1–3 dots/cell; 2 = 4–10 dots/cell and/or very few dot clusters; 3 = >10 dots/cell and/or <10% positive cells have dot clusters; 4 = >10 dots/cell and/ or >10% positive cells have dot clusters. For statistical analyses a dichotomized score was used: 0 (low) or 1–4 (high). Investigators MI and SK evaluated the slides with high inter-reader agreement (k = 0.85). For discordant cases (*n* = 10) a final score was assigned following a consensus from both investigators. *CYP27A1* mRNA expression was evaluable in 194 tumors.

### Survival analyses endpoints

The impact of CYP27A1 expression on prognosis was evaluated for three distinct clinical endpoints: (1) RFS, defined as the time from BC diagnosis to any event of invasive loco-regional, distant metastasis or death from any cause, (2) DRFS, defined as the time from BC diagnosis to occurrence of the first distant metastasis or death from any cause, and (3) OS, defined as the time from BC diagnosis to death from any cause.

### Cell lines, reagents, and cell culture

The ER+ BC cell lines T47D and MCF7 were obtained from the American Type Culture Collection and maintained in RPMI-1640 (HyClone^TM^, Cat# SH3060501.01) and DMEM-F12 media (HyClone^TM^, Cat# SH30271.01), respectively, supplemented with 10% FBS (HyClone^TM^) and an antibiotic cocktail made of 100 U/ml penicillin and 100 mg/ml streptomycin. Cells were grown in a culture medium supplemented with lipid-depleted fetal calf serum (LDS) (Sigma, Cat# S5394) for experimental treatments. 27HC (Avanti polar lipids, Cat# 700021 P) and 17 β-estradiol (Sigma–Aldrich, Cat# E8875) were reconstituted in DMSO and ethanol, respectively.

### 27HC treatment and sulforhodamine B (SRB) proliferation assay

To assess the impact of 27HC on BC cell proliferation in vitro, T47D and MCF7 cells were treated with 1 µM 27HC in a lipid-depleted medium with or without estradiol supplementation. Cells were pre-cultured in 12-well cell culture plates (30,000 cells/well for T47D and 20,000 cells/well for MCF7) in phenol red-free medium supplemented with charcoal-stripped fetal bovine serum (HyClone, Cat# SH30068) for 48 h to phase out any residual effects due to estrogen signaling from the cell maintenance medium. Next the cells were exposed to a medium supplemented with LDS and containing 1 μM 27HC with or without the addition of 1 nM estradiol. After 72 h of culture under the respective treatments, cells were fixed overnight in 10% trichloroacetic acid at 4 °C, washed five times with tap water, and air-dried. Cells were stained for 15 min with SRB (0.4% w/v in 1% acetic acid), washed five times with acetic acid, and then air-dried. The SRB stain was dissolved with TrisBase for 10 min and absorbance was measured at 572 nm using an ELISA plate reader. Differences in proliferation were calculated relative to the vehicle treated control cells. All experiments were repeated three times. Results are presented as the mean ± standard deviation (SD).

### Quantitative real-time PCR

Cells were exposed for 48 h to the same treatment conditions described for the proliferation assay (1 µM 27HC in lipid-depleted medium with or without 1 nM estradiol supplementation), after which total RNA was isolated using the RNeasy mini kit (Qiagen, Cat# 74104) according to the manufacturer’s instructions. cDNA was synthesized from 1 µg of total RNA using a High-Capacity cDNA Reverse Transcription kit (Applied Biosystems, Cat# 4368814). qRT-PCR was performed using the TaqMan QuantiTect Probe PCR Kit (Qiagen, Cat# 204343) together with predesigned primer/probe sets (Applied Biosystems) to amplify TFF1 (Hs00907239_m1), PgR (Hs01556702_m1), LDLR (Hs01092525_m1), and ABCA1 (Hs01059118_m1). Expression of all transcripts, as threshold cycle (Ct) value, was measured in triplicate and normalized to the housekeeping gene, GAPDH (Hs99999905_m1). Levels of mRNA expression in treated cells compared to untreated cells were determined by the 2-ΔΔCt method. All experiments were performed twice.

### Statistical analyses

**Fig. 5:**
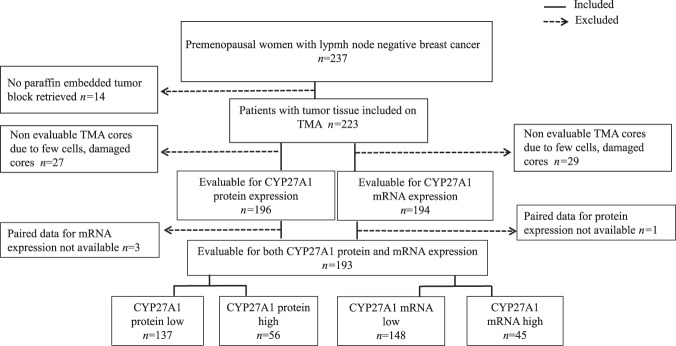
CONSORT diagram of the study. Flowchart showing numbers of patients included in and excluded from the analyses.

Cohen’s kappa coefficient (κ) was used to evaluate inter-reader agreement for CYP27A1 protein and mRNA expression in tumors. Overall, tumors from 193 patients were evaluable for both CYP27A1 protein and mRNA expression. These constitute the final population included in the analyses presented in this study (Fig. 5). The concordance between CYP27A1 protein and mRNA expression in the tumor was evaluated using Spearman’s correlation coefficient. Associations of CYP27A1 and other tumor pathological features were tested using chi-square tests. Associations of CYP27A1 with prognosis were analyzed by Kaplan–Meier analyses and Cox-regression yielding log-rank *P* values and hazard ratios (HR) with 95% confidence intervals (CI), respectively. Multivariable Cox regression models were adjusted for age at diagnosis (continuous variable), ER status (positive vs negative), HER2 status (positive vs negative) tumor size (≤20 mm vs >20 mm), NHG (I & II vs III), Ki67 status (low ≤ 20% vs high > 20%), radiotherapy (yes vs no), endocrine therapy (yes vs no), and chemotherapy (yes vs no). All analyses for associations between tumor characteristics and survival are exploratory and have not been corrected for multiple testing. *P* values reported are from two-sided statistical tests and *P* < 0.05 was statistically significant.

For study power calculations including 200 patients, of which 25% had a high intratumoral expression of CYP27A1 and with a median survival time of 19 years for patients with low intratumoral CYP27A1 expression, it was possible to detect true HRs of ≤ 0.54 or ≥ 2.06 with a probability (power) of 0.8. The type I error probability associated with this test of the null hypothesis that the experimental and control survival curves are equal is 0.05. The power calculations were performed with the PS Power and Sample Size Calculation Program, version 3.1.2

For assessing the statistical significance of respective in vitro treatments on cell proliferation and differential gene expression, one-way and two-way ANOVAs, respectively, were performed with post hoc comparisons using Dunnett’s method to adjust for multiple testing.

Significance levels are represented by *P* < 0.05(*) and *P* < 0.01(**).

### Reporting summary

Further information on research design is available in the Nature Research Reporting Summary linked to this article.

## Supplementary information

Supplementary Information

Reporting Summary

## Data Availability

Patient data used for survival analyses are not publicly available to protect patient privacy according to data privacy rules. Enquiries regarding access to data regarding the current study should be directed to the corresponding author.
