# Supplementary material for: High CYP27A1 expression is a biomarker of favorable prognosis in premenopausal patients with estrogen receptor positive primary breast cancer
Source: NPJ Breast Cancer. 2021 Sep 23;7:127. doi: 10.1038/s41523-021-00333-6 (PMC8460751; doi:10.1038/s41523-021-00333-6)
Supplement: Supplementary file 1 — Supplementary Information [file 41523_2021_333_MOESM1_ESM.pdf]

Supplementary Table 1: Patients and tumor characteristics for all patients included in the SB91B study (n=237) and in cases included (n=193) and excluded (n=44) in the analyses presented in the current study

	**All Patients with Invasive breast cancer (n=237)**	**Patients with evaluable CYP27A1 (n=193)**	**Patients with non evaluable CYP27A1 (n=44)**
**Characteristic**	n (%)	n (%)	n (%)
**Age at BC diagnosis, yrs**			
Median	47	47	48
Range	29-57	29-56	32-57
**Tumor Size, mm**			
≤ 20 mm	174 (73.4)	144 (74.6)	30 (68.2)
> 20 mm	63 (26.6)	49 (25.4)	14 (31.8)
**Nottingham histological grade**			
I	73 (30.8)	58 (30.1)	15 (34.1)
II	82 (34.6)	70 (36.3)	12 (27.3)
III	72 (30.4)	62 (32.1)	10 (22.7)
**ER status**			
Positive	156 (65.8)	128 (66.3)	28 (63.6)
Negative	81 (34.2)	65 (33.7)	16 (36.4)
**PgR status**			
Positive	168 (70.9)	138 (71.5)	30 (68.2)
Negative	69 (29.1)	55 (28.5)	14 (31.8)
**HER2 status**			
Positive	23 (11.0)	23 (12.8)	0 (0)
Negative	187 (89.0)	156 (87.2)	31 (100.0)
Missing	27	14	13
**Ki67, %**			
≤20	136 (68)	115 (66.1)	21 (80.8)
>20	64 (32)	59 (33.9)	5 (19.2)
Missing	37	19	18
**Endocrine therapy**			
Yes	8 (3.4)	8 (4.1)	0 (0)
No	229 (96.6)	185 (95.9)	44 (100)
**Chemotherapy**			
Yes	21 (8.9)	18 (9.3)	3 (6.8)
No	216 (91.1)	175 (90.7)	41 (93.2)
**Radiotherapy**			
Yes	117 (49.4)	98 (50.8)	19 (43.2)
No	120 (50.6)	95 (49.2)	25 (56.8)

Supplementary Table 2: Prognostic impact of differential CYP27A1 protein and mRNA expression

		Hazard ratio (95% CI)	
	**No at risk**	**Model[a*]**	**Model [b**]**
**Recurrence-free survival**			
**mRNA-/ protein-**	102	Ref	Ref
**mRNA+/ protein-**	15	0.39 (0.13-1.13)<br>*P* = 0.08	0.35 (0.12-1.05)<br>*P* = 0.06
**mRNA-/ protein+**	21	0.46 (0.18-1.20)<br>*P* = 0.11	0.44 (0.17-1.15)<br>*P* = 0.09
**mRNA+/ protein+**	26	0.28 (0.11-0.71)<br>*P* = 0.007	0.28 (0.11-0.71)<br>*P* = 0.007
**Distant recurrence free survival**			
**mRNA-/ protein-**	102	Ref	Ref
**mRNA+/ protein-**	15	0.15 (0.02-1.11)<br>*P* = 0.06	0.14 (0.02-1.08)<br>*P* = 0.06
**mRNA-/ protein+**	21	0.44 (0.13-1.49)<br>*P* = 0.19	0.44 (0.13-1.48)<br>*P* = 0.19
**mRNA+/ protein+**	26	0.25 (0.08-0.76)<br>*P* = 0.01	0.27 (0.08-0.84)<br>*P* = 0.02
**Overall survival**			
**mRNA-/ protein-**	102	Ref	Ref
**mRNA+/ protein-**	15	0.13 (0.02-0.96)<br>*P* = 0.04	0.13 (0.02-0.94)<br>*P* = 0.04
**mRNA-/ protein+**	21	0.75 (0.31-1.84)<br>*P* = 0.53	0.74 (0.30-1.82)<br>*P* = 0.52
**mRNA+/ protein+**	26	0.23 (0.08-0.70)<br>*P* = 0.009	0.23 (0.08-0.71)<br>*P* = 0.01

*Model a : Model adjusted for age at diagnosis, tumor size, Ki67, ER, HER2 and Nottingham histological grade.
**Model b : Model a + adjusted for local (radiotherapy) and systemic (endocrine and chemotherapy) treatment.

Supplementary Table 3: Prognostic impact of the joint expression of tumor proliferation markers with CYP27A1 expression

	Hazard ratio (95% CI)		
	**Univariable**	**Model[a *]**	**Model[b**]**
**Distant recurrence free survival**			
CYP27A1 low/ NHG I+II	Ref	Ref	Ref
CYP27A1 low/ NHG III	2.43 (1.32-4.45)<br>*P* = 0.004	1.45 (0.57-3.69)<br>*P* = 0.43	1.47 (0.59-3.68)<br>*P* = 0.41
CYP27A1 high/ NHG I+II	0.41 (0.09-1.70)<br>*P* = 0.22	0.21 (0.03-1.6)<br>*P* = 0.13	0.23 (0.03-1.76)<br>*P* = 0.16
CYP27A1 high/ NHG III	0.62 (0.22-1.76)<br>*P* = 0.36	0.38 (0.11-1.38)<br>*P* = 0.14	0.40 (0.11-1.45)<br>*P* = 0.16
CYP27A1 low/ Ki67 low	Ref	Ref	Ref
CYP27A1 low/ Ki67 high	2.50 (1.34-4.64)<br>*P* = 0.04	1.26 (0.51-3.11)<br>*P* = 0.61	1.24 (0.50-3.11)<br>*P* = 0.64
CYP27A1 high/ Ki67 low	0.19 (0.03-1.42)<br>*P* = 0.11	0.19 (0.02-1.40)<br>*P* = 0.10	0.19 (0.03-1.46)<br>*P* = 0.11
CYP27A1 high/ Ki67 high	0.64 (0.22-1.85)<br>*P* = 0.41	0.35 (0.09-1.31)<br>*P* = 0.12	0.37 (0.09-1.39)<br>*P* = 0.14

*Model a : Model adjusted for age at diagnosis, tumor size, Ki67, ER, HER2, Nottingham histological grade

**Model b : Model a + adjusted for local (radiotherapy) and systemic (endocrine and chemotherapy) treatment
