# Supplementary material for: High CYP27A1 expression is a biomarker of favorable prognosis in premenopausal patients with estrogen receptor positive primary breast cancer
Source: NPJ Breast Cancer. 2021 Sep 23;7:127. doi: 10.1038/s41523-021-00333-6 (PMC8460751; doi:10.1038/s41523-021-00333-6)
Supplement: Supplementary file 2 — Reporting Summary [file 41523_2021_333_MOESM2_ESM.pdf]

nature research

Corresponding author(s): Siker Kimbung

Last updated by author(s): Aug 17, 2021

Reporting Summary

Nature Research wishes to improve the reproducibility of the work that we publish. This form provides structure for consistency and transparency in reporting. For further information on Nature Research policies, see our Editorial Policies and the Editorial Policy Checklist.

Statistics

For all statistical analyses, confirm that the following items are present in the figure legend, table legend, main text, or Methods section.

n/a	Confirmed	
☐	☒	The exact sample size (*n*) for each experimental group/condition, given as a discrete number and unit of measurement
☐	☒	A statement on whether measurements were taken from distinct samples or whether the same sample was measured repeatedly
☐	☒	The statistical test(s) used AND whether they are one- or two-sided<br>*Only common tests should be described solely by name; describe more complex techniques in the Methods section.*
☐	☒	A description of all covariates tested
☐	☒	A description of any assumptions or corrections, such as tests of normality and adjustment for multiple comparisons
☐	☐	A full description of the statistical parameters including central tendency (e.g. means) or other basic estimates (e.g. regression coefficient) AND variation (e.g. standard deviation) or associated estimates of uncertainty (e.g. confidence intervals)
☒	☐	For null hypothesis testing, the test statistic (e.g. *F*, *t*, *r*) with confidence intervals, effect sizes, degrees of freedom and *P* value noted<br>*Give P values as exact values whenever suitable.*
☒	☐	For Bayesian analysis, information on the choice of priors and Markov chain Monte Carlo settings
☒	☐	For hierarchical and complex designs, identification of the appropriate level for tests and full reporting of outcomes
☐	☒	Estimates of effect sizes (e.g. Cohen's *d*, Pearson's *r*), indicating how they were calculated

Our web collection on statistics for biologists contains articles on many of the points above.

Software and code

Policy information about availability of computer code

Data collection	N/A
Data analysis	Statistical Analyses were performed in SPSS version 26

For manuscripts utilizing custom algorithms or software that are central to the research but not yet described in published literature, software must be made available to editors and reviewers. We strongly encourage code deposition in a community repository (e.g. GitHub). See the Nature Research guidelines for submitting code & software for further information.

Data

Policy information about availability of data

All manuscripts must include a data availability statement. This statement should provide the following information, where applicable:

- Accession codes, unique identifiers, or web links for publicly available datasets
- A list of figures that have associated raw data
- A description of any restrictions on data availability

Patient data used for survival analyses are not publicly available to protect patient privacy according to data privacy rules.

Field-specific reporting

Please select the one below that is the best fit for your research. If you are not sure, read the appropriate sections before making your selection.

☒ Life sciences ☐ Behavioural & social sciences ☐ Ecological, evolutionary & environmental sciences

For a reference copy of the document with all sections, see nature.com/documents/nr-reporting-summary-flat.pdf

Life sciences study design

All studies must disclose on these points even when the disclosure is negative.

Sample size	For study power calculations including 200 patients, of which 25% had a high intratumoral expression of CYP27A1 and with a median survival time of 19 years for patients with low intratumoral CYP27A1 expression, it was possible to detect true HRs of ≤0.54 or ≥2.06 with a probability (power) of 0.8. The type I error probability associated with this test of the null hypothesis that the experimental and control survival curves are equal is 0.05. The power calculations were performed with the PS Power and Sample Size Calculation Program, version 3.1.2
Data exclusions	Our study is based on a prospective cohort of 237 pre-menopausal women presenting with node negative breast cancer in the South of Sweden between 1991-1994.
Replication	In vitro experiments were run in duplicates and repeated at least twice.
Randomization	No randomisation was required in this study.. However analyses were performed comparing effects between different patient subgroups and experimental conditions.
Blinding	During assessment of biomarker status by IHC and RNAscope all investigators were blinded to other patient and tumor characterisitcs.

Reporting for specific materials, systems and methods

We require information from authors about some types of materials, experimental systems and methods used in many studies. Here, indicate whether each material, system or method listed is relevant to your study. If you are not sure if a list item applies to your research, read the appropriate section before selecting a response.

Materials & experimental systems

n/a	Involved in the study	
☐	☒	Antibodies
☐	☒	Eukaryotic cell lines
☒	☐	Palaeontology and archaeology
☒	☐	Animals and other organisms
☐	☒	Human research participants
☒	☐	Clinical data
☒	☐	Dual use research of concern

Methods

n/a	Involved in the study	
☒	☐	ChIP-seq
☒	☐	Flow cytometry
☒	☐	MRI-based neuroimaging

Antibodies

Antibodies used	anti-CYP27A1 rabbit monoclonal antibody , Abcam, EPR7529, ab126785
Validation	This antibody has been thoroughly validated for application in western blotting and immunohistochemistry by using protein lysates from cell lines and paraffin-embedded cell line pellets and primary breast tumors. Cell lines used for validation included HEPG2 cells, a panel of both ER+ and ER- BC cell lines. The expression in human liver tissue was used as a positive control during validation. Positive cells showed granular cytoplasmic reactivity by IHC. A blocking peptide (ab139504) was able to ablate the signal. In immuno blotting, the antibody reacted to a clean single band at the predicted molecular weight. References: PMID: 24288332, PMID: 33176848, PMID: 28130224, PMID: 28442559

Eukaryotic cell lines

Policy information about cell lines

Cell line source(s)	Cell lines were directly purchased from the vendor American Type Culture Collection
Authentication	Cell lines were not authenticated in house. We only used early passages (<20) of cell lines in our experiments.
Mycoplasma contamination	All cell lines were tested quarterly for mycoplasma contamination. Samples were routinely submitted to eurofins genomics to

Mycoplasma contamination	perform standardised qPCR test for mycoplasma under ISO17025 accreditation , and no contamination with mycoplasma was detected.
Commonly misidentified lines (See ICLAC register)	n/a

Human research participants

Policy information about studies involving human research participants

Population characteristics	Our study is based on a prospective cohort of 237 pre-menopausal women presenting with node negative breast cancer in the South of Sweden between 1991-1994.
Recruitment	Patients were included in a prospective clinical trial (SB91B) conducted to validate the prognostic value of an index based on tumor proliferation (S-phase fraction), PgR status, and tumor size. All patients provided their informed consent to be included in the SB91B clinical trial.
Ethics oversight	The study was approved by the ethics committee of Lund University Hospital (LU 240-01).

Note that full information on the approval of the study protocol must also be provided in the manuscript.
